# Microplastics in the Olfactory Bulb of the Human Brain

**DOI:** 10.1001/jamanetworkopen.2024.40018

**Published:** 2024-09-16

**Authors:** Luís Fernando Amato-Lourenço, Katia Cristina Dantas, Gabriel Ribeiro Júnior, Vitor Ribeiro Paes, Rômulo Augusto Ando, Raul de Oliveira Freitas, Ohanna Maria Menezes M. da Costa, Renata S. Rabelo, Kelly Cristina Soares Bispo, Regiani Carvalho-Oliveira, Thais Mauad

**Affiliations:** 1 Institute of Biology, Freie Universität Berlin, Berlin, Germany; 2 Department of Pathology, Sao Paulo Medical School, University of São Paulo, São Paulo, Brazil; 3 Brazilian Synchrotron Light Laboratory (LNLS), Brazilian Center for Research in Energy and Materials (CNPEM), São Paulo, Brazil; 4 Department of Fundamental Chemistry, Institute of Chemistry, University of São Paulo (IQUSP), São Paulo, Brazil

## Abstract

**Importance:**

Microplastic (MP) pollution is an emerging environmental and health concern. While MPs have been detected in various human tissues, their presence in the human brain has not been documented, raising important questions about potential neurotoxic effects and the mechanisms by which MPs might reach brain tissues.

**Objective:**

To determine the presence of MPs in the human olfactory bulb and to analyze their characteristics such as size, morphology, color, and polymeric composition.

**Design, Setting, and Participants:**

This case series study used a cross-sectional design involving the analysis of olfactory bulb tissues obtained from deceased individuals during routine coroner autopsies. The sampling procedures were conducted at São Paulo City Death Verification Service, with laboratory analysis carried out at the Brazilian Synchrotron Light Laboratory (LNLS). Participants included 15 adult individuals who had been residents of São Paulo for more than 5 years and underwent coroner autopsies. Exclusion criteria included previous neurosurgical interventions. Data analysis was performed in April 2024.

**Exposure:**

The primary exposure assessed was the presence of MPs in the olfactory bulb, analyzed through direct tissue examination and digested tissue filtration followed by micro-Fourier transform infrared spectroscopy.

**Main Outcomes and Measures:**

The main outcomes were the identification and characterization of MPs within the olfactory bulb, including their size, morphology, color, and polymeric composition.

**Results:**

The median age of the 15 deceased individuals was 69.5 years, ranging from 33 to 100 years, with 12 males and 3 females. MPs were detected in the olfactory bulbs of 8 out of 15 individuals. A total of 16 synthetic polymer particles and fibers were identified, with 75% being particles and 25% being fibers. The most common polymer detected was polypropylene (43.8%). Sizes of MPs ranged from 5.5 μm to 26.4 μm for particles, and the mean fiber length was 21.4 μm. Polymeric materials were absent in procedural blank and negative control filters, indicating minimal contamination risk.

**Conclusions and Relevance:**

This case series provides evidence of MPs found in the human olfactory bulb, suggesting a potential pathway for the translocation of MPs to the brain. The findings underscore the need for further research on the health implications of MP exposure, particularly concerning neurotoxicity and the potential for MPs to bypass the blood-brain barrier.

## Key Points

**Question:**

Can microplastics reach the olfactory bulb in the human brain?

**Findings:**

This case series analyzed the olfactory bulbs of 15 deceased individuals via micro-Fourier transform infrared spectroscopy and detected the presence of microplastics in the olfactory bulbs of 8 individuals. The predominant shapes were particles and fibers, with polypropylene being the most common polymer.

**Meaning:**

The presence of microplastics in the human olfactory bulb suggests the olfactory pathway as a potential entry route for microplastics into the brain, highlighting the need for further research on their neurotoxic effects and implications for human health.

## Teaser

This case series investigates microplastics in the olfactory bulbs of deceased individuals and examines their size, morphology, color, and polymeric composition.

## Introduction

The ubiquity of microplastic (MP) pollution has become a pervasive environmental concern,^1^ raising questions about its occurrence within the human body and its harmful effects.^2^ While MPs have been detected in various organs of the human body, such as the lungs,^3,4^ large and small intestines,^5^ liver,^6^ placenta,^7,8^ semen,^9^ and bloodstream,^10^ to our knowledge, there have been no published studies to date reporting their presence in the human brain.

The presence of the blood-brain barrier (BBB) is likely an important limiting factor for the access of MPs to the human brain via hematogenous translocation. Despite this, some animal studies have shown that MPs can impair the BBB and reach the brain via oral ingestion, leading to neurotoxic effects.^11,12,13^ Another potential entry site for micro- and nanoplastics (MNPs) in the human brain is the olfactory pathway.^14^ This pathway involves olfactory neurons in the nasal that transmit information about odors to the central olfactory system of the brain. Olfactory axons pass through the cribriform plate (CP) of the ethmoid bone and reach the olfactory bulbs (OB), which are connected to the limbic system of the brain.

There are different levels of evidence suggesting that the olfactory pathway might allow the translocation of exogenous particles to the brain. Environmental black carbon particles have been detected in various human brain regions, with one of the highest concentrations found in the OB, measuring 420.8 particles/mm^3^.^15^ Rarely, the 15- to 30-μm–sized ameboid form of Naegleria fowleri penetrates the brain via the nose, causing amebic meningoencephalitis.^16^ Affected individuals typically present with the disease after contact with contaminated freshwater bodies or after rinsing the nose with nonsterile tap water.^17^ Furthermore, the permeability of this barrier has been evoked as a possible quicker and safer drug delivery route to the brain,^18,19^ as well as access to cerebrospinal fluid through nasal lymphatic vessels.^20^

In this study, given the ubiquitous presence of MPs in the air^21^ and their previous identification in the human nasal cavity,^22,23^ we hypothesized that the smallest-size fraction of MPs could reach the OB. Therefore, we conducted an investigation into the presence of MPs within human OB obtained from 15 deceased individuals during coroner autopsies. We identified and analyzed various characteristics of the MPs, including their size, morphology, color, and polymeric composition.

## Methods

This case series study was approved by the ethical board of the São Paulo University Medical School, in compliance with the Helsinki Declaration. Written informed consent was provided by the deceased individuals’ next of kin. The study was conducted from February 2023 to May 2024 and followed the Reporting Guideline for Case Series.^46^

### Study Population

We obtained the bilateral OBs from 15 adult individuals who underwent routine coroner autopsies at the São Paulo City Death Verification Service of University of São Paulo to determine the cause of death. All individuals had been residents of São Paulo for more than 5 years. Cases in which the deceased had previously undergone neurosurgical interventions were not selected for the study. Information regarding previous occupations and underlying diseases was obtained through questionnaires administered to the next of kin. Additionally, autopsy reports were reviewed. We also collected samples from the OB of 2 stillbirths at 7 months gestation, as a negative control for the study. The collection of OBs took place between February 2023 and February 2024.

### Quality Control and Quality Assurance and Evaluation of Sample Processing

We implemented a plastic-free approach to safeguard the integrity of our results. This strategy facilitated a thorough assessment of potential sources of variability and error, thereby enhancing the reliability of our collected data. All procedures, from the OB sampling to the micro-Fourier transform infrared (μFTIR) spectroscopy analysis, followed the protocols recommended by several studies.^24,25,26^ Briefly, all solutions were prefiltered through a Whatman cellulose filters with a mesh size of 0.45 μm. Stainless steel materials, glassware, and samples were covered with aluminum foil (before and after processing) to avoid airborne sample contamination. Ultrapure water with a resistivity of 18.2 mΩ was obtained from a Milli-Q purification device (Millipore Corp). Glass and stainless-steel materials were washed thoroughly using the purified water 3 times and then using acetone P.A. to remove any particles or fibers that have adhered to the glass. The scientific staff responsible for handling samples wore exclusively 100% cotton laboratory coats and were required to remove any plastic or textile bracelets, rings, and watches to minimize the risk of sample contamination. Clean latex gloves were used for all procedures. The samples were processed in a clean laminar flow cabinet (ISO class 5, SKU330313, Hipperquímica, SP, Brazil). Blank filters (47 mm) were used from the OB collection to the sample filtering to assess possible airborne contamination. A clean filter was also used as a negative control. Access to the μFTIR spectroscopy and the digestion/filtration room was restricted to the operators only, to avoid air flow in the room and the suspension/resuspension of possible atmospheric contaminants.

### Sample Processing

The presence of MPs in the OB was assessed in 2 ways: directly on the tissue and a digested assessment. The cryo-cuts method preserves the spatial context of MPs within the tissue, allowing their proximity to anatomical structures such as blood vessels to be observed. This is crucial for understanding potential pathways of MPs translocation and accumulation within the OB. The digestion method ensures that MPs that are deeply embedded in the tissue are not overlooked. Postdigestion, MPs are concentrated on filters, which can then be analyzed for a more accurate quantification and identification without interference from the tissue matrix. By combining these 2 methods, the study maximized the probability of detecting and characterizing MPs within the OB.

#### OB Cryo-Cuts

The left OB of each case was horizontally cryo-sectioned using a Leica CM1860 UV cryostat (Leica Biosystems) at 10 μm thickness and thaw-mounted onto 5 mm × 5 mm gold/chromium-coated silicon dioxide/silicon substrates. No fixatives were used for the tissue sections. The samples were then freeze-dried for 48 hours (Freezone 6 [Labconco Corp]) and examined by optical microscopy (Eclipse LV100ND [Nikon Instruments Inc]). The freeze-drying process maintains the integrity of biological tissues by extracting water without substantially compromising their structure. Futhermore, the presence of water molecules, characterized by strong hydrogen bonding, poses a considerable challenge in FTIR measurements, as they mask specific signals indicative of chemical compositions.^27^

The procedures took place in a biosafety level 2 room in the Cryogenic Preparations Laboratory (LCRIO) at the Brazilian Synchrotron Light Laboratory (LNLS), National Center for Energy and Materials Research (CNPEM).

#### Sample Digestion and Filtering

Immediately after sampling, the right OBs from 10 selected cases were individually frozen at −20 °C in glass vials, covered with aluminum foil, and sealed with a glass lid until the digestion. For 5 patients, there was no available tissue for digestion. The tissues were then incubated for 12 hours at 40 °C using the enzyme mixture Corolase 7089 (20 UHb/mL)^4^ inside the laminar flux hood.

The solution was then filtered using a glass vacuum filtration system (Sigma-Aldrich) and silver membrane filters (25 mm in diameter and 0.45 microns pore size [Millipore]). Subsequently, the filters were kept individually in closed Petri dishes inside a glass dissector until the spectroscopy analysis. Due to the material characteristics, a recovery test was not feasible.

### Micro-Fourier Transform Infrared Spectroscopy

We performed single-point μFTIR microspectroscopy measurements in reflection mode using a diffraction-limited IR microscope (Cary 620 [Agilent Technologies]). The IR microscope is coupled to a Michelson interferometer responsible for the frequency demultiplexing of the mid-IR broadband response. We used a 1000 K Globar source and illumination and interferograms detection was done by using a high-sensitivity cryo-cooled Mercury–Cadmium-Telluride (MCT [Infrared Associates Inc]). After the interferometer, the IR beam was directed to a 25 × objective that produced an illumination spot of 420 μm × 420 μm on the sample’s surface. This field of view was further reduced to 50 to 100 μm by slits to concentrate the analysis around specific particles. The reflected light was collected through a confocal arrangement by the same objective lens and then directed to the MCT detector. FTIR spectra were generated by calculating the Fourier transforms of the recorded interferograms. The spectral resolution was configured at 16 cm^−1^, encompassing the range from 4000 to 700 cm^−1^. Each μFTIR spectrum was normalized to the spectrum of a clean gold surface, which served as a reference background. The cryo-cuts and digested filters were fully analyzed. The μFTIR analyses took place in the IMBUIA beamline at the Brazilian Synchrotron Light Laboratory (LNLS), National Center for Research in Energy and Materials (CNPEM).

The acquired spectra were processed manually using the KnowItAll Informatics System 2024 (John Wiley and Sons Inc). The comparative analysis was performed with the help of FTIR spectra libraries developed for MPs research, including the FTIR Library of Plastic Particles (FLOPP),^28^ FTIR Library of Plastic Particles Sourced from the Environment (FLOPP-e),^28^ siMPLe database,^29^ and KnowItAll IR Spectral Library. We adopted a Hit Quality Index greater than 75% of agreement between characteristic bands of polymers observed in reference materials with bands observed in unknown particles or fibers.^30,31^

### Microphotograph Analysis

We determined particle sizes by analyzing microphotographs obtained through μFTIR spectroscopy. ImageJ 1.54g software (US National Institutes of Health) was used for accurate measurements.

### Statistical Analysis

Descriptive analyses were performed using SPSS Statistics 26.0 software (IBM Inc). These analyses were performed in April 2024.

## Results

The median (range) age of the 15 deceased individuals was 69.5 (33-100) years. They included 12 males and 3 females. Demographic information is detailed in Table 1. Apart from the 2 cases with histological evidence of previous ischemic cerebral infarction and 1 case with a subarachnoid hematoma due to a ruptured aneurysm of the middle cerebral artery, there were no cerebral histological abnormalities in the remaining cases. The mean (SD) mass of the OB (left or right) was 0.187 (0.050) g, ranging from 0.100 to 0.273 g.

**Table 1.** Demographic and Autopsy Findings of the Decedents

Decedent	Demographic data			Underlying diseases	Cause of death
	Age at death, y	Sex	Occupation		
1	69	Male	Driver	Lung cancer	Pulmonary tuberculosis
2	76s	Female	Cook	Systemic arterial hypertension	Acute pulmonary edema
3	70	Male	Mechanic	Acute myocardial infarction, prostate cancer	Pulmonary edema
4	33	Male	Freelancer	Epilepsy, chronic alcoholism	Pneumonia
5	71	Male	Waiter	Ruptured thoracic aortic dissection, systemic arterial hypertension, chronic alcoholism	Hemothorax
6	78	Male	Manager	Atherosclerosis	Acute myocardial infarction
7	65	Male	Tinker	Pleural empyema, systemic arterial hypertension	Septic shock
8	71	Female	Cook	Middle cerebral artery aneurysm, systemic arterial hypertension, neuralgia	Subarachnoid hemorrhage
9	62	Male	Tinker	Systemic arterial hypertension, chronic alcoholism	Acute myocardial infarction
10	77	Male	Bricklayer	Ischemic heart disease, systemic arterial hypertension, diabetes, chronic alcoholism	Acute pulmonary edema
11	57	Male	Unemployed	Systemic arterial hypertension, heart disease, chronic alcoholism, congenital muscular dystrophy	Unknown
12	70	Female	Pensioner	Hypertrophic heart disease, asthma	Pulmonary edema
13	66	Male	Cleaner	Hypertensive heart disease, systemic arterial hypertension, chronic alcoholism	Pulmonary edema
14	78	Male	Concierge	Stroke, Chagas disease	Bronchopneumonia
15	100	Male	Tinker	Myeloid leukemia	Bronchopneumonia

A total of 16 synthetic polymer particles and fibers were identified in 8 out of the 15 deceased individuals, with a range from 1 to 4 MPs per OB. Of these, 75% were particles, of which 83.4% were fragments and 16.6% were spheres, while 25% were fibers with a length-to-width ratio exceeding 3. The particles had a mean (SD) length of 12.1 (7.2) μm, ranging from 5.5 to 26.4 μm, and a mean (SD) width of 8.9 (6.4) μm, ranging from 3.0 to 25.4 μm. The fibers exhibited a mean (SD) length of 21.4 (2.6) μm, ranging from 19.0 to 24.5 μm, and a mean (SD) width of 3.8 (1.8) μm, ranging from 3.0 to 6.0 μm.

In the procedural blank filters, we detected 2 cotton fibers, 2 silica beads, and 1 silicate fragment. Polymeric materials were absent in both the procedural blank and negative control filters. From the 2 collected samples in stillborn, we were able to analyze 1 case, which did not show the presence of MPs. The other case had insufficient material for analysis.

Polypropylene was the most prevalent polymer (43.8%), followed by polyamide, nylon, and polyethylene vinyl acetate (12.5%). This was followed by polyethylene, perlon polyamide, and wool-polypropylene, which accounted for 6.3%). Upon comparison with the reference spectral library of plastic materials, the identified MP particles and fibers exhibited indications of weathering. The μFTIR spectra of the weathered MPs differed substantially from those of pristine standard samples; multiple peaks in the spectra of weathered MPs were attenuated or entirely absent.

**Figure 1.**
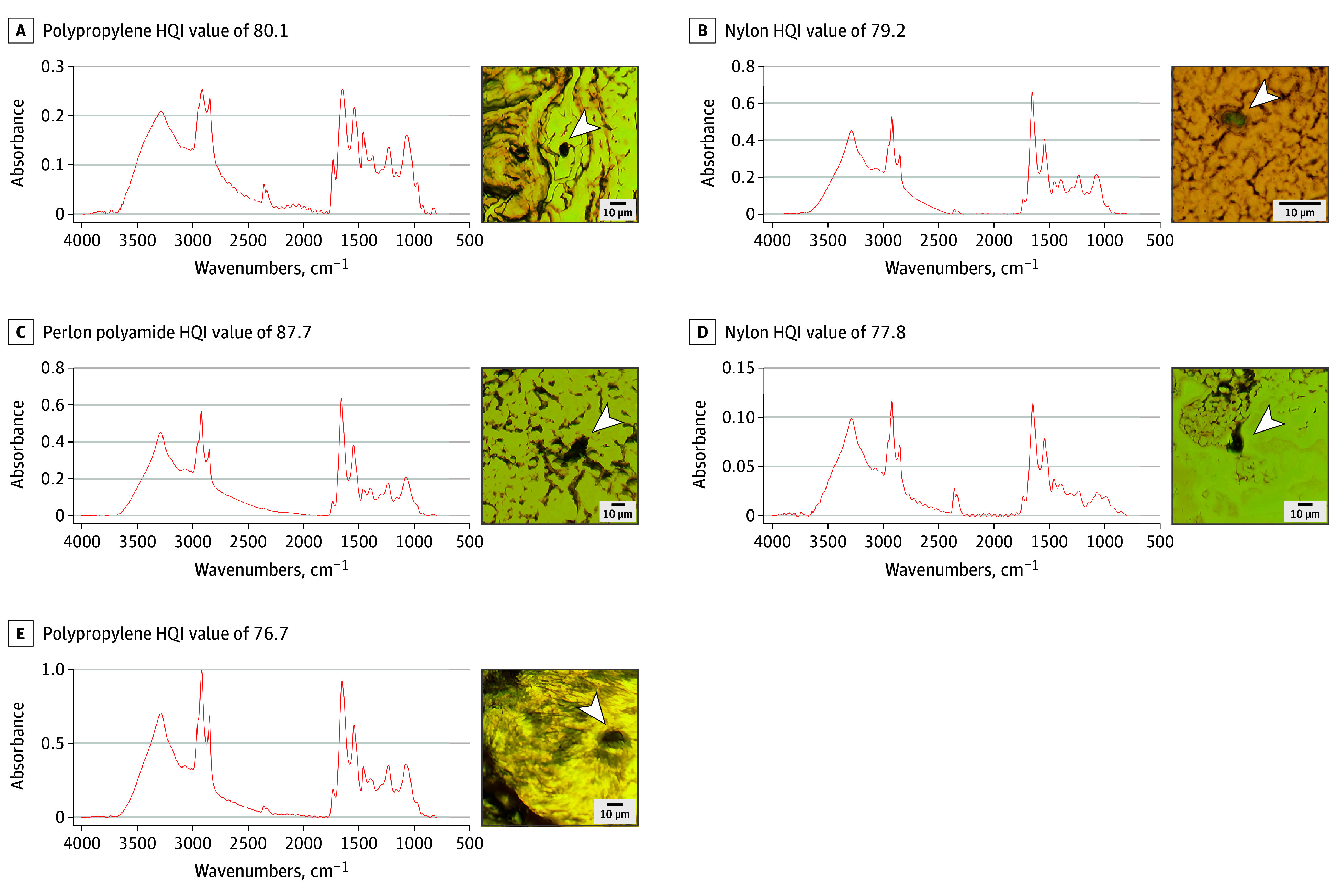
Microphotographs and Micro-Fourier Transform Infrared (μFTIR) Spectra of the Microplastics Found in the Olfactory Bulb Tissue HQI indicates hit quality index.

**Figure 2.**
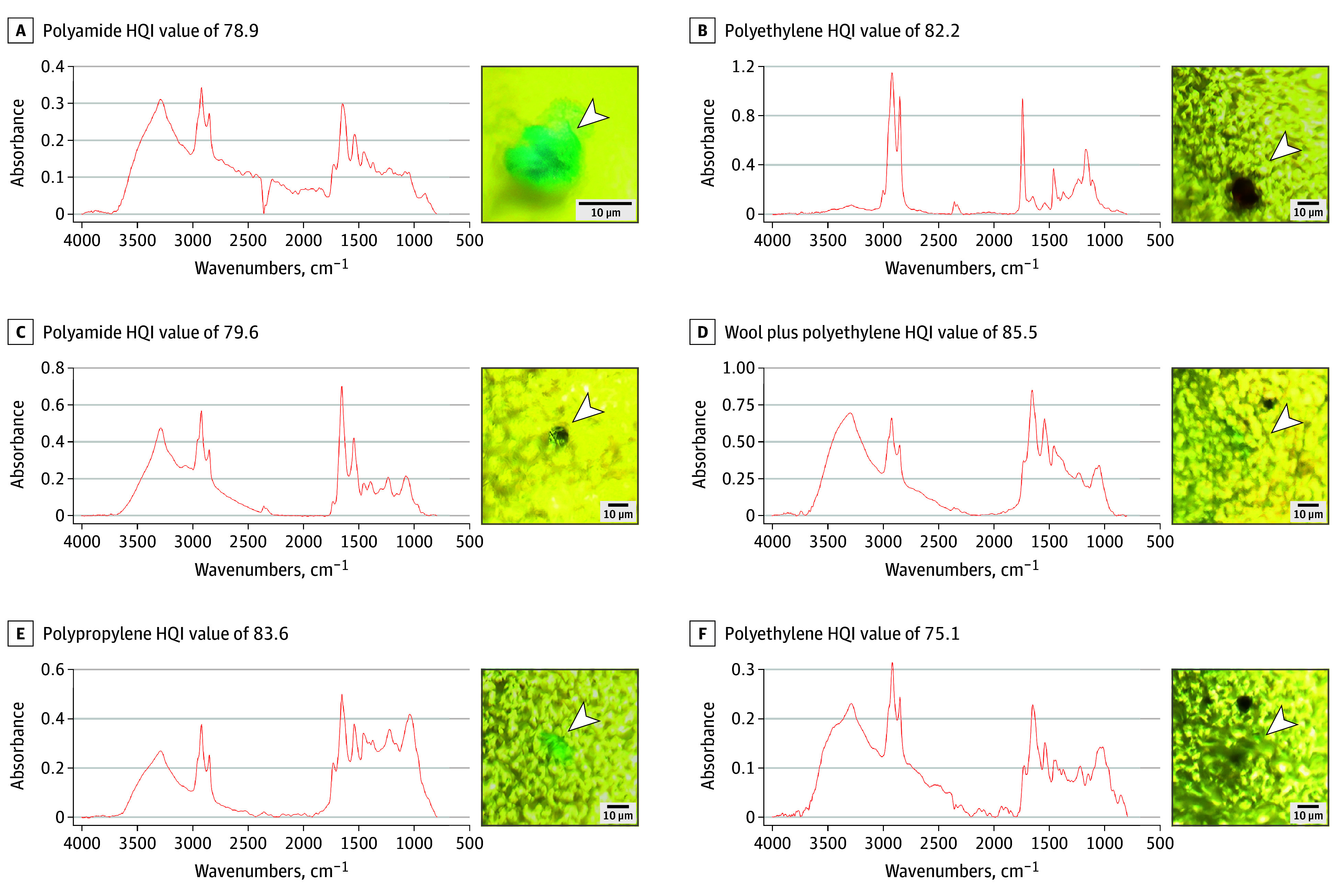
Microphotographs and Micro-Fourier Transform Infrared (μFTIR) Spectra of the Main Microplastics Found in the Digested Olfactory Bulb HQI indicates hit quality index.

Microphotographs and μFTIR point-spectra showing the main types of MP detected in the OB are shown in Figure 1 and Figure 2. The complete μFTIR point-spectra results of the digested OB are presented in the eFigure in Supplement 1. Table 2 provides details regarding the morphology, color, and chemical characterization of the particles and fibers.

**Table 2.** Morphology and Polymeric Matrix of the Identified Particles and Fibers

Decedent	Cryo-cuts (left OB)				Digested Filters (right OB)			
	Format	Size, μm^a^	Polymer matrix	Color	Format	Size, μm^a^	Polymer matrix	Color
1	Particle	5.5 × 5.1	Polypropylene	Black	Particle	13.6 × 8.9	Polyamide	Blue
2	Particle	13.4 × 5.0	Nylon	Green	NA	NA	NA	NA
5	Particle	8.5 × 4.0	Perlon polyamide	Gray	NA	NA	NA	NA
6	NA	NA	NA	NA	Particle	18.1 × 10.6	Polyethylene vinyl acetate	White
	NA	NA	NA	NA	Fiber	19.6 × 6.0	Polypropylene	Green
	NA	NA	NA	NA	Particle	15.2 × 8.6	Polyethylene vinyl acetate	Brown
	NA	NA	NA	NA	Fiber	24.5 × 2.6	Wool + polypropylene	Green
7	NA	NA	NA	NA	Particle	26.4 × 25.4	Polypropylene	Blue/gray
	NA	NA	NA	NA	Particle	17.0 × 9.1	Polypropylene	Blue/gray
	NA	NA	NA	NA	Fiber	19.0 × 4.4	Polypropylene	Pink
11	NA	NA	NA	NA	Particle	8.3 × 7.0	Polyamide	Gray
12	Particle	6.6 × 3.0	Polypropylene	Gray	Particle	5.9 × 4.4	Polyethylene	Light blue
					Particle	19.2 × 16.7	Polypropylene	Green
15	Fiber	22.6 × 3.0	Nylon	Blue	NA	NA	NA	NA

Abbreviations: NA, not applicable; OB, olfactory bulb.

^a^ Particles are listed as length × width; fibers are listed as length × diameter.

## Discussion

To our knowledge, this is the first study in which the presence of MPs in the human brain was identified and characterized using μFTIR, allowing quantification and characterization of the morphology and polymeric matrix. Specifically, we detected particles as the predominant shape in the OB in 8 out of 15 individuals who underwent autopsy in Sao Paulo. Our data extend the notion that not only black carbon^15^ but also MP accumulate in the OB in humans.

We believe that the anatomy of the cribriform plate of the ethmoid bone may serve as a gateway in the nasal passages from within the skull. This plate, situated between the frontal and sphenoid bones, lies horizontally and contains multiple foramina, each less than 1 mm in diameter.^32^ The OB lies directly above it, and the olfactory neurons of the nasal mucosa reach the OB via the foramina of the cribriform plate. Recent studies have shown that part of the cerebrospinal fluid outflow occurs via lymphatic vessels that surround the olfactory axons, reaching the nasal mucosa and extending toward the nasal lymphoid tissue.^33^ Ossification of the CP occurs by 1 year of age,^34^ and the total area of the perforations is age-dependent; it is 3.79 to 3.99 mm^2^ in those over 50 years of age and 5.61 to 7.91 mm^2^ in those under 50 years of age. This decrease in the area over time, causing compression and dysfunction of the olfactory nerves, is thought to explain the decreased olfactory sensation in older individuals.^35^ Furthermore, in mice, paracellular spaces in the olfactory epithelium can reach 5 to 20 μm in the medial-lateral dimension of the transport and a 10- to 100-μm range observed in the rostral-caudal dimension.^36^ If a similar situation is observed in humans, this could represent another factor facilitating entry of larger particles in the brain via the cribriform plate.

Given the widespread presence of MPs in the air, some of which are associated with PM_2.5_,^37^ the identification of MPs in the nose^45^ and now in the OB, along with the vulnerable anatomical pathways, reinforces the notion that the olfactory pathway is an important entry site for exogenous particles to the brain. In previous epidemiological studies, exposure to PM_2.5_ has been associated with neurological and psychiatric adverse outcomes, such as dementia.^38,39^ Some neurodegenerative diseases, such as Parkinson disease, seem to have a connection with nasal abnormalities as initial symptoms.^40^ In experimental studies, both exposures to PM_2.5_ and MPs have shown to cause several neurotoxic effects, including disturbances on the brain development.^41,42^ The cribriform plate reaches maturation at 1 to 2 years of age, which is a critical time window during which MP penetration into the brain could have negative effects on the organ maturation.

In this study, the MP polymeric matrix found in the OB corresponds to the most produced and manufactured plastics, such as polypropylene, nylon/polyamide, polyethylene and polyethylene vinyl acetate, present in packaging, clothes and home accessories, suggesting indoor environments as a major source of inhaled MPs.^21,43^

### Limitations

This study has certain limitations. Although the olfactory pathway seems a likely exposure route, we cannot dismiss the possibility of multiple entry routes. MPs might have reached the OB either through systemic circulation, crossing the BBB, or via the respiratory pathway through the trigeminal nerve.^44^ The biologic matrix of the OB tissues can be a confounding factor when analyzing MP spectra due to its similarity to some polymeric materials. Therefore, we were cautious to consider suspect particles as polymeric material only when spectral bands highly matched with weathered bands from MP libraries (HQI >75%). In the filtered samples, the biological matrix was previously digested, not being an issue. Given the maximum spatial resolution (3 μm) of μFTIR spectroscopy setup and the limited capacity of analysis for other techniques, we were unable to detect nanoplastics. It is likely that the number of plastics in the submicron range with the potential to cause substantial biological damage would be far more numerous.

Avoiding contamination is one of the biggest challenges when analyzing MP. Due to the presence of MP fibers and particles in the air, we have used blank samples in all methodological procedures to detect contamination of the air. We found no MP in our procedural blanks, which supports the validity of our results. Furthermore, we had the opportunity to analyze the brains of 2 stillbirths. However, the status of brain tissue maceration made the analysis challenging due to difficulties in sampling and processing.

## Conclusions

This case series describes the presence of MPs in the OB, mainly particles of the most commonly produced/processed polymers for clothing and packaging such as polypropylene and nylon. Our data support the idea that the olfactory pathway is an important entry site for environmental air pollutants. Considering the potential neurotoxic effects caused by MPs in the brain, and the widespread environmental contamination with plastics, our results should raise concern in the context of increasing prevalence of neurodegenerative diseases. Noninvasive imaging technologies, such as magnetic resonance imaging, are needed to overcome the current limitations in tissue analysis of different human organs and to improve the understanding of the health hazards of MPs.

## Notes

Supplement 1. **eFigure.** Microphotographs and µFTIR Spectra of the Microplastics Found in the Digested Olfactory Bulb

Supplement 2.
Data Sharing Statement
