# Supplementary material for: Microplastics in the Olfactory Bulb of the Human Brain
Source: JAMA Netw Open. 2024 Sep 16;7(9):e2440018. doi: 10.1001/jamanetworkopen.2024.40018 (PMC11406405; doi:10.1001/jamanetworkopen.2024.40018)
Supplement: Supplement 1. — eFigure. Microphotographs and µFTIR Spectra of the Microplastics Found in the Digested Olfactory Bulb [file jamanetwopen-e2440018-s001.pdf]

Supplemental Online Content

Amato-Lourenço LF, Dantas KC, Júnior GR, et al. Microplastics in the olfactory bulb of the human brain. *JAMA Netw Open.* 2024;7(9):e2440018. doi:10.1001/jamanetworkopen.2024.40018

eFigure. Microphotographs and µFTIR Spectra of the Microplastics Found in the Digested Olfactory Bulb

This supplemental material has been provided by the authors to give readers additional information about their work.

© 2024 Amato-Lourenço LF et al. *JAMA Network Open.*

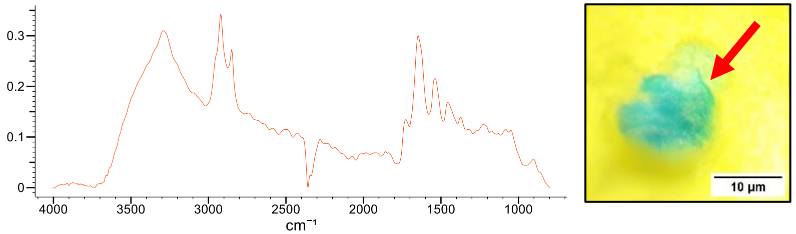

Polyamide – HQI: 78.9

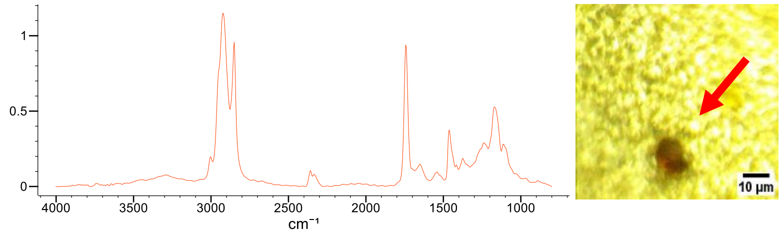

Polyethylene vinyl acetate – HQI: 82.2

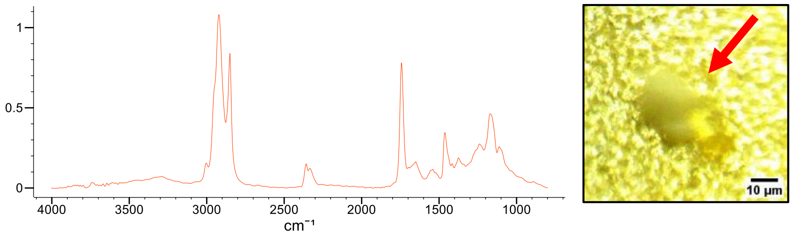

Polyethylene vinyl acetate – HQI: 84.1

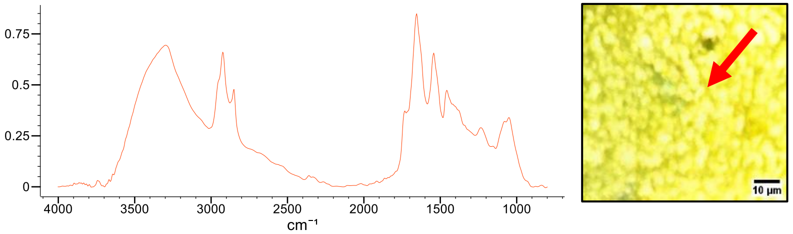

Wool + Polypropylene – HQI: 85.5

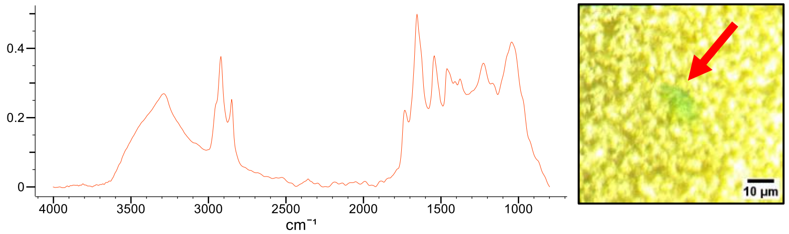

Polypropylene – HQI: 83.6

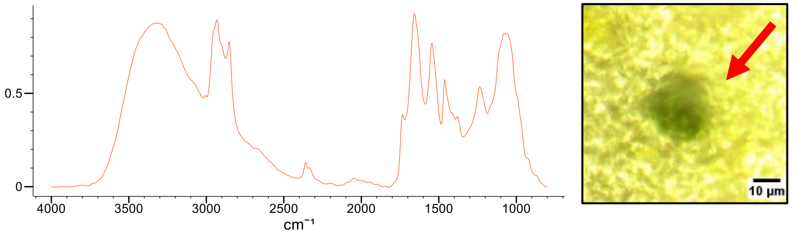

Polypropylene – HQI: 83.1

© 2024 Amato-Lourenço LF et al. *JAMA Network Open.*

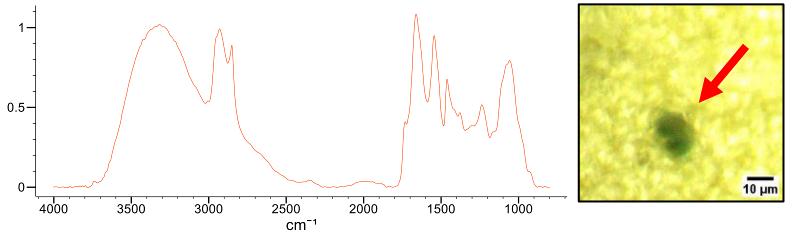

Polypropylene – HQI: 81.0

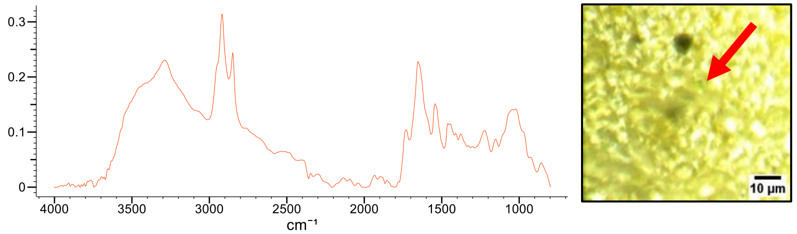

Polyethylene – HQI: 75.1

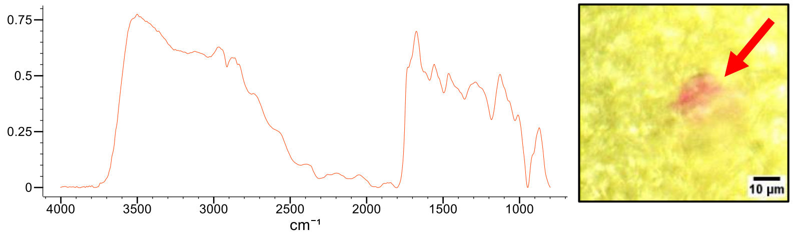

Polypropylene – HQI: 83.4

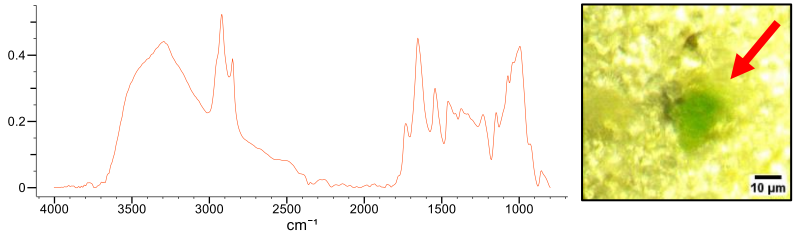

Polypropylene – HQI: 81.8

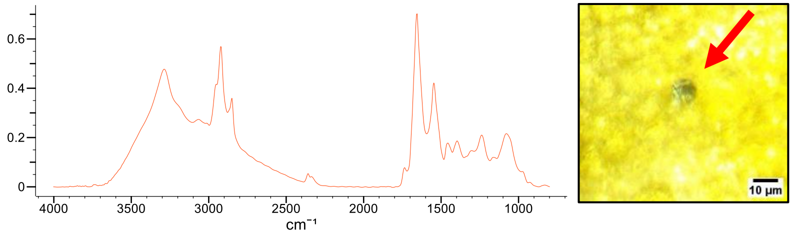

Polyamide – HQI: 79.6

eFIGURE - Microphotographs and µFTIR spectra of the microplastics found in the digested olfactory bulb. Bar scale – 10 µm. HQI = hit quality index value.

© 2024 Amato-Lourenço LF et al. *JAMA Network Open.*
