# Supplementary material for: Microplastics in the Olfactory Bulb of the Human Brain
Source: JAMA Netw Open. 2024 Sep 16;7(9):e2440018. doi: 10.1001/jamanetworkopen.2024.40018 (PMC11406405; doi:10.1001/jamanetworkopen.2024.40018)
Supplement: Supplement 2. — Data Sharing Statement [file jamanetwopen-e2440018-s002.pdf]

Data Sharing Statement

Amato-Lourenço. Microplastics in the Olfactory Bulb of the Human Brain. *JAMA Netw Open*. Published September 16, 2024. doi:10.1001/jamanetworkopen.2024.40018

Data
Data available: Yes
Data types: Participant data with identifiers, Data (not involving human participants)
How to access data: Data or materials can be obtained from the corresponding author upon request
When available: With publication

Supporting Documents
Document types: Informed consent form
How to access documents: Data or materials can be obtained from the corresponding author upon request
When available: With publication

Additional Information
Who can access the data: researchers whose proposed use of the data has been approved
Types of analyses: any purpose
Mechanisms of data availability: after approval of a proposal
